# *Chlamydia trachomatis ompA* Genotype and Clinical Signs of Trachoma in a Longitudinal Tanzanian Cohort

**DOI:** 10.3390/pathogens15070705

**Published:** 2026-07-04

**Authors:** Anna J. Harte, Elias Mafuru, Athumani Ramadhani, Tamsyn Derrick, Harry Pickering, Tara Mtuy, Patrick Massae, Ehsan Ghasemian, Aiweda Malissa, Robin L. Bailey, David C. W. Mabey, Matthew J. Burton, Martin. J. Holland

**Affiliations:** 1 Clinical Research Department, Faculty of Infectious Tropical Diseases, London School of Hygiene and Tropical Medicine, Keppel Street, London WC1E 7HT, UK; 2 Kilimanjaro Christian Medical Centre, Moshi P.O. Box 3010, Tanzania

**Keywords:** *Chlamydia trachomatis*, trachoma, genovar, genovariant, MOMP, Tanzania, mass drug administration, *ompA*, antigenic variation, vaccine development

## Abstract

Trachoma, caused by *Chlamydia trachomatis* (*Ct*), persists as a major cause of preventable blindness despite the global SAFE strategy. Understanding how *Ct* genovars and genovariants influence infection dynamics and clinical outcomes is crucial for sustaining elimination efforts and informing vaccine development. A four-year longitudinal study was conducted in a trachoma-endemic region of Tanzania across multiple rounds of mass drug administration (MDA) with azithromycin. *Ct* infections were genotyped by ompA sequencing to identify genovars and genovariants. Associations between genetic variants, bacterial load, and clinical signs of trachoma were assessed. Following MDA, a shift in *Ct* genovar prevalence occurred from genovar B to genovar A. Genovar B was associated with more severe clinical signs, including follicles, papillae, and scarring, whereas genovar A infections exhibited higher bacterial loads. Among 121 individuals with recurrent infections, 94% were re-infected with the same genovar, indicating limited protective immunity and incomplete clearance despite MDA coverage exceeding 60%. The genovariants B2, B9, and A2 predominated, with an A → T amino acid substitution in B9 potentially modifying antigenic recognition. Post-MDA, normalized genovariant diversity increased, suggesting ongoing transmission or strain reintroduction. Distinct genovar-associated clinical and immunological patterns underscore the need to elucidate genovar-specific virulence and immune evasion mechanisms. These findings provide key insights for optimizing trachoma control and advancing vaccine development.

## 1. Introduction

*Chlamydia trachomatis* (*Ct*) is an obligate intracellular bacterium that causes trachoma, an infection of the eye that can lead to blindness [1]. It is estimated that trachoma is responsible for visual impairment in 1.9 million people worldwide, with 103 million people living in trachoma-endemic areas [2]. Although *Chlamydia trachomatis* has been widely studied, there are currently no approved vaccines against infection or the development of blinding complications. There are, however, vaccines being developed with primary targets focusing on the Major Outer Membrane Protein (MOMP), a chlamydial protein that expresses multiple T and B cell epitopes [3].

MOMP is encoded by the gene outer membrane protein A (*ompA*), and it is sequence differences in this gene that enable different chlamydial serotypes to be identified. There are 19 recognized serotypes of *Ct*, of which serotypes A-C are overwhelmingly responsible for ocular infection. Ocular serotypes display a level of geographical localisation, with serotypes A and B dominating in Africa, Asia, and South America, and serotypes Ba and C were mostly identified in indigenous communities in Australia and islands in Australasia [4]. The 19 serotypes can be divided into 2 main immunotypes known as the B- and C-complexes. These are dependent on the four major epitope regions present in MOMP, known as variable domains (VD) 1–4. Serotypes A and C are part of the C-complex, whereas serotype B (and the minor serotype Ba) are part of the B-complex. This difference in complex-type between the two dominant ocular serotypes A and B is significant, as vaccines developed against only one complex may lack protection against infection with the other [5].

Due to this key role in interacting with the immune system, it is postulated that *Ct* may benefit from slight differences in MOMP, which help to evade the immune response and hinder the development of subsequent immunity [6]. However, while numerous ocular *ompA* genovariants have been identified [7,8,9,10], there is limited evidence that demonstrates whether this translates to either greater evasion of the immune system or enhanced infectiousness or virulence. One of the challenges in determining the role of sequence genovariants within *Ct* genotypes is that many studies rely solely on cross-sectional data. This approach captures a single snapshot in time, allowing researchers to observe an association between specific sequence genovariants and the presence of active trachoma. However, because cross-sectional studies can only suggest associations, these are insufficient to establish a causal link, account for the impact of recurrent infections, or determine how specific genotypes influence the progression to conjunctival scarring. Therefore, longitudinal studies that track individuals over time are necessary to fully elucidate long-term causal effects.

In this study, samples were collected from a longitudinal cohort of children living in a trachoma-endemic region of Tanzania [11,12,13,14]. A cohort of 666 children from three villages in northern Tanzania was initially enrolled in a 4-year study of progressive scarring trachoma, infection, and clinical signs, with samples and trachoma grades recorded every 3 months. Annual mass drug administration with azithromycin was delivered as recommended by the WHO. The identification of *Ct*-positive children and associated clinical grading in a longitudinal study provides a valuable opportunity to investigate the *ompA* sequence genovariants that may be associated with the clinical signs of trachoma, recurrent infection, and the effect of MDA.

## 2. Materials and Methods

### 2.1. Ethics

Ethical approval for the collection of the samples used in this study was granted by the London School of Hygiene & Tropical Medicine and the Tanzania National Institute for Medical Research, Kilimanjaro Christian Medical University College, as outlined in the original study plan [14]. As all participants were under the legal age of consent, written informed consent from a parent or legal guardian was necessary for enrolment, with the study explained in local languages prior to requesting consent.

### 2.2. Study Design, Population, and Treatment

The study design, population, and treatment protocols have been previously described by Ramadhani et al. [11,12,14]. In brief, 666 children aged 6–10 years living in three rural villages in Northern Tanzania were eligible for enrollment, of whom 616 were initially enrolled. Data collection spanned 2012–2016, targeting sample collection every three months (17 timepoints total). At each timepoint, some children were not examined due to absence from the village, relocation, or declining participation. The median number of participants seen across the 17 timepoints was 451 (range: 380–536). The cumulative loss to follow-up (approximately 33%; range 20–43%) is within the expected 30–50% range for long-term community-based studies with high respondent burden. A total of 7640 conjunctival swabs underwent *Ct* diagnostic testing. Antibiotic administration followed the Tanzanian National Trachoma Control Programme guidelines to manage the community bacterial reservoir. Annual mass drug administration (MDA) with azithromycin included all community members over 6 months of age; participants received a single oral dose (1 g for adults; 20 mg/kg for children). Infants under 6 months received a supervised six-week regimen of tetracycline eye ointment (twice-daily). Each village received three annual MDA rounds (between timepoints 3–4, 7–8, and 11–12), with Village 3 receiving a fourth round between timepoints 15 and 16 due to trachoma prevalence remaining above 15%.

### 2.3. Sample Collection, Processing, and Ct Diagnostics

Conjunctival swab samples and clinical trachoma grades were collected following the method described by Ramadhani et al. 2019 [12]. The key clinical signs recorded at each timepoint were conjunctival scarring, follicles, and papillae, each scored on a scale of 0–3 [15]. Scarring scores were determined using the method outlined in Ramadhani et al. [11]. In brief, photographic grading was used to assess new or progressive conjunctival scarring by comparing baseline and final images. When baseline or final images were missing, the nearest timepoints were substituted (timepoints 2 and 16). All images were graded by an experienced ophthalmologist using a detailed scarring system [16]. The collected samples were stored at −80 °C until ready for processing. DNA extraction and diagnostic qPCR were performed in Tanzania and the UK on all samples to determine *Ct* positivity and bacterial load as previously described [14]. *Ct* was detected in the timepoint 1 samples using a droplet digital PCR assay (ddPCR) and at all other timepoints by multiplex quantitative real-time PCR (qPCR) previously evaluated against ddPCR [17,18]. Both assays detect chlamydial plasmid open reading frame 2 (*pORF2*), *Ct* outer membrane complex protein B (*omcB*), and human endogenous control gene ribonuclease P/MRP Subunit P30 (*RPP30*), using the same primer and probe sequences (Table 1). The ddPCR reaction contained 5 μL of DNA template and primers/probes at a final concentration of 0.3 nM using Taqman mastermix. PCR reaction conditions were as follows: 95 °C for 10 min, then 40 cycles of 95 °C for 10 s, 60 °C for 30 s, and finally 98 °C for 12 min. Droplets were then examined for fluorescence on a QX200TM Droplet Reader (Bio-Rad, Watford, UK), providing a quantitative result. The qPCR assay was performed on a ViiA7 thermal cycler (Thermo Fisher Scientific, Paisley, UK.) using TaqMan Multiplex Master mix in a final volume of 20 μL, containing 4 μL of DNA template and primers and probes each at a final concentration of 0.3 nM. Cycling conditions were as follows: 95 °C hold for 20 s followed by 40 cycles of 95 °C for 1 s and 60 °C for 20 s. Samples were tested in duplicate and were considered *Ct*-positive if either replicate amplified *omcB* and/or *pORF2* with a cycle threshold (CT) value < 40. Sample sufficiency and PCR inhibition were determined by the use of an internal positive control, *RPP30*. *RPP30* CT values < 40 with clear evidence of amplification were regarded as sufficient and used to confirm a blank *omcB* or *pORF2* result as a true negative (*Ct* not detected) rather than a failed swab (insufficient human cells collected, extraction failure, or PCR inhibition). All diagnostic assays included *Ct* PCR-positive (DNA extracted from cultured Human Epithelial type-2 (HEp-2) cells, which had been infected with *Ct* strain A2497 and the absolute quantity determined by ddPCR) and negative controls, as well as routine laboratory extraction “air” controls. In addition, during diagnostic testing, both laboratories participated in external quality control molecular diagnostics programmes (Quality Control in Molecular Diagnostics (QCMD—www.qcmd.org)), demonstrating satisfactory standards in testing.

### 2.4. OmpA Genotyping

The *ompA* sequences from the cohort were obtained in 2 ways, as previously described [19,20]. Firstly, the *ompA* sequence was extracted from the *Ct* whole-genome sequences (WGS) using a reference-based assembly method. In brief, sequences were extracted from quality-filtered reads by aligning to three reference genomes (A/Har13, B/Jali20, and C/TW3) with Bowtie2. The *ompA* sequence with the lowest percentage-missing calls per whole-genome sequence was used in downstream analyses. Secondly, PCR-positive *Ct* samples not selected for WGS due to insufficient yield were subjected to an *ompA*-nested PCR for amplicon-based Sanger sequencing. Table 2 shows the PCR and sequencing primers. PCR was conducted using a Veriti Thermocycler (Thermo Fisher). For the first round, 20 μL of 5 prime MasterMix, 20 μL of molecular-grade water, and 2.5 μL each of forward and reverse primers with 5 μL of *Ct*-positive sample template DNA were cycled: 2 min at 94 °C, then 35 cycles of 94 °C for 15 s and 62 °C for 75 s, and a final elongation step of 72 °C for 10 min. This PCR resulted in a product of 1077 base pairs (b.p) in size. Five μL of the sample was resolved by gel electrophoresis, and if no band was present, nested PCR was performed. For the second round of PCR, 10 μL of 5 prime MasterMix (QuantaBio, Beverly, MA, USA) and 1.25 μL of forward and reverse primers were used with 11.5 μL molecular-grade water added to 1 μL of a 1/200 dilution of the product from the first round. Second round cycling conditions were: 2 min at 94 °C, then 35 cycles of 94 °C for 15 s and 65 °C for 75 s, and a final elongation step of 72 °C for 10 min. Samples were tested for PCR product by gel electrophoresis. The PCR reactions were then cleaned before sequencing using a ratio of 0.8× final volume of AMPure XP magnetic beads (Beckman Coulter, Amersham, UK), following the manufacturer’s instructions and quantified on the Qubit 2.0 Fluorometer (Thermo Fisher). PCR products were then diluted to a concentration of ~10 ng/μL for Sanger sequencing at Source Bioscience (Cambridge, UK) and sequenced with the *ompA*-87 and *ompA*-1059 primers. The Sanger nucleotide sequences and the WGS genome *ompA*-extracted sequences generated in this study are available at PZ438546–PZ438994. The *Ct* whole-genome sequence data from which the *ompA* sequence was extracted is available from the ENA accession PRJEB46956.

### 2.5. Genovariant Calling

Sequence files received from Source Bioscience were first base-called and trimmed to remove low quality bases. The resulting high-quality sequences were then converted to the FASTA format and subsequently used to query all *Chlamydia* sequences available in NCBI via BLASTn IBLAST + 2.15.0) to determine the genotype/genovar/genovariant according to the consensus nomenclature. Sequencing data for every sample was then split into their respective genotypes and aligned to a reference sequence: B-TW/5 (M17342) for genotype B and A/HAR-13 (DQ064279) for genotype A to determine genovar. All sequences were trimmed to 850 bp in length to ensure a standardized read, with the trimmed sequence spanning full coverage of VDII-VDIII, and partial coverage of VDI and VDIV. Sequences where there were overlapping peaks at specific positions were re-sequenced to confirm the overlap, and if true overlapping peaks were found, genovariants could not be called, and these sequences were not included in any genovariant analyses.

### 2.6. Antigenicity Prediction

The antigenicity of the top two genotype B genovariants was compared using the Immune Epitope Database (IEDB.org) B cell epitope prediction online tool. The semi-empirical Kolaskar and Tongaonkar Antigenicity Epitope Prediction method was used [21], with a 281 amino acid peptide broken down into sequentially overlapping 7-mer peptides. Data are shown as antigenicity scores, with values above 1.0 indicating an above-average likelihood of antigenicity.

### 2.7. Data Analysis

Basic exploratory tests were done to compare between genovars, genovariants, and pre- and post-MDA. The difference in the loads of the plasmid *pORF2* gene between the three genovars was tested using linear mixed-effects models to account for repeated measures present for some individuals, with ID set as the random effect. The load between genovar B and the most frequent genovariant (B9) was tested using the same method. The difference between the clinical signs of follicles, papillae, and scarring between pre- and post-MDA timepoints was tested using the non-parametric Kruskal–Wallis test. Association analyses were conducted using multiple response variables. The original ordinal clinical scores of scarring, follicles, and papillae were analyzed separately using mixed-effects ordinal logistic regression in R (CLMM package 2023.12-5). Each positive infection was treated as a separate event, with the role of the dependent variables of age, gender, genotype, *Ct* load, and genovariant tested on each clinical sign, with balozi and id used as the random effects. Only one genovariant (B9) had a high enough allele frequency to perform a statistical analysis in relation to clinical signs. Models were run, including age and gender as standard, with females set as the reference level for gender; the variables genotype, *Ct* load, and genovariant were tested using a backwards selection approach, with the model with the lowest Akaike information criterion (AIC) chosen for the final estimates. Two new response variables were created to investigate the influence of the dependent variables on the presence of multiple clinical signs, following the technique outlined in Chin et al. [9]. The first was defined as no scarring = 0, scarring but no inflammation (inflammation defined as papillae score of 3) = 1, and scarring and inflammation = 2. FPC grades of P3 equate to a diagnosis of TI by the WHO’s simplified system. The same was done using the follicle score in place of scarring, but including both F2 and F3 follicle scores. These response variables were also analyzed using ordinal mixed-effects logistic regression. Scarring progression was categorized as a binomial variable and analyzed using mixed-effects logistic regression using the package lme4 [22], including the dependent variables age and gender, and a binary variable representing the presence or absence of a serial genovar infection, defined as an individual experiencing serial genovar infections with a differing *ompA* genovar or *ompA* genovariant over the 4-year study period. The pathogen diversity of each village was calculated for pre- and post-MDA timepoints and first and last timepoints using the Shannon index from the Vegan package 2.6-4 [23] in each village.

## 3. Results

**Figure 1:**
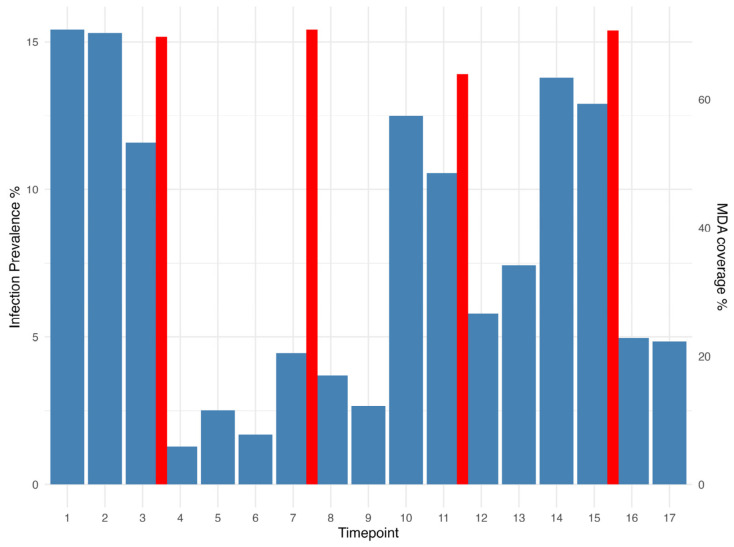
*Chlamydia trachomatis* prevalence in the Tanzanian cohort of children confirmed by diagnostic PCR, based on the number of positives in the number of children sampled at each timepoint. The red bars show the percentage of MDA coverage for the study cohort at each treatment point. The fourth red bar between timepoints 15 and 16 represents treatment for village 3 only.

A total of 593/7856 (7.7%) specimens were identified as positive for *Ct* infection by diagnostic qPCR, with 449 (75%, 237 individuals) of these successfully sequenced for *ompA*. The prevalence of infection at baseline was 15.4%, with a final prevalence of 4.8% at timepoint 17, 4 years after baseline [12] (Figure 1). Of the people successfully sequenced for *ompA*, 121 were positive for *Ct* infection at two or more timepoints, 17 of which had different genotypes (A, B, or Ba) at separate timepoints.

### 3.1. Genovars

**Figure 2:**
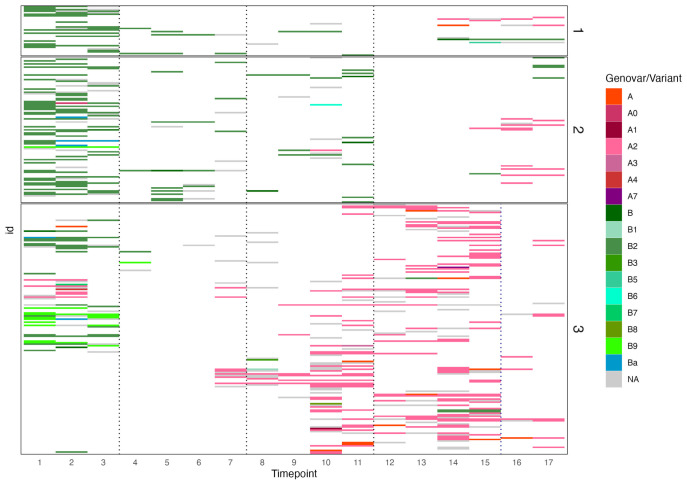
The number and genovar/variant ID of *Chlamydia trachomatis* positive people at each timepoint. Dotted lines indicate where the population underwent a mass drug administration event. Each row of the plot represents an individual, with all individuals grouped by village (1, 2. 3). NA indicates samples that tested positive but could not be successfully sequenced. Legend entries without a number after the serovar denote sequences where the variant could not be determined.

**Figure 3:**
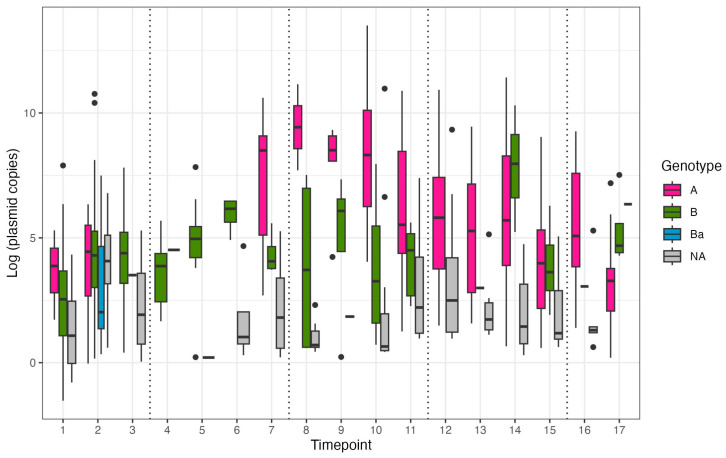
Log_10_ *Chlamydia trachomatis* plasmid load per genotype at each timepoint for all three Tanzanian villages. Dashed lines indicate mass drug administration. The fourth dotted line between timepoints 15 and 16 represents treatment for village 3 only. NA indicates samples that tested positive but could not be successfully sequenced.

Three genovars were found in these samples: genovar A (*n* = 220), genovar B (*n* = 222), and the minor genovar Ba (*n* = 7). The majority of samples at the first three timepoints prior to the first round of MDA were genovar B (Figure 2). Most genotype A infections were found in village 3, whereas genovar B was more evenly spread. The number of *Ct*-positive individuals dropped substantially after the first MDA, with a prevalence of 1% at timepoint 4. The majority (94%) of infections prior to any treatment were genovar B (168 B/Ba; 10 A), whereas in the years following the first round of MDA, genovar A accounted for 78% of infections (61 B; 213 A). While the proportion of genovar A individuals increased over time from timepoint 7 onwards, low numbers of genovar B were maintained throughout most of the remaining timepoints (Figure 2). The analysis of bacterial load demonstrated that genovar A infections (mean load: 12,370 copies/μL) had a statistically higher load relative to B/Ba genotypes overall (mean load: 754 copies/μL) (*t*-test, *p* < 0.01). Genovar load varied substantially over time and in response to MDA treatment (Figure 3).

### 3.2. Genovariants

Within a genovar, five A genovariants and eight B genovariants were identified (Supplementary Tables S1 and S2), relative to the respective reference genotypes for A and B. Single-nucleotide variants (SNVs) were only defined as single-nucleotide polymorphisms when more than 1% of the population expressed the SNV. There were no genovariants in the genovar Ba sequences. Genovariants were located throughout the sequence, with one of the dominant genovar A genovariant (A2) SNPs located three AAs from a known MOMP immunogenic epitope (VAGLEK) located in VD 1 [24]. For genovar A, genovariant A2 accounted for over 97% of the samples; all other genovariants had a frequency of one. For genovar B, there were two genovariants with a frequency above one, genovariant B2 (*n* = 197) and genovariant B9 (*n* = 21). Genovariant B9 was found in predominantly pre-MDA timepoints 1–3, with one individual in timepoint 4. There was no significant difference between the loads of the most common genovariant B2 and genovariant B9.

All genovar A samples, but one, had the two base pair changes shown in genovariant A2, relative to the reference sequence A/HAR13 (Supplementary Table S1). The first of these changes resulted in an amino acid change from alanine to threonine, and the second changed the amino acid from isoleucine to leucine. All B genotype samples had the same genovariant changes relative to the reference sequence B/TW-5, shown in genovariant B2. Genovariant B9, the most common genovariant (*n* = 21), had just one SNP at the very last position in variable domain 4, which changed the amino acid from alanine to threonine. Predicted antigenicity indicated that the change in amino acid between the most common genovariant B2 (GDVKTSA) and the second most common genovariant B9 (GDVKTST) resulted in a small reduction in antigenicity on the Kolaskar and Tongaonkar scale (1.005 to 0.983). For the most common genovar A genovariant (A2), relative to the A/HAR13 reference, the amino acid changed from alanine to threonine and resulted in a reduction in antigenicity from 1.086 to 1.064.

### 3.3. Clinical Signs Association Analyses

Genovar B showed a significant association with the presence of follicles, papillae, and scarring across all timepoints. A Kruskal–Wallis test comparing each clinical sign between timepoints sorted into two groups of pre-MDA (timepoints 1–3) and post-MDA (timepoints 4–17) showed that there was a strong significant difference between the two MDA stages (*p* < 0.001) for all three clinical signs. Age was also indicated as significant for the decreased risk of developing follicles but increased risk of scarring, with increasing age increasing the likelihood of scarring by a factor of 1.2, whereas increasing age decreased the likelihood of follicles (Table 3).

For the new variables created following Chin et al. [9], age and genotype were significant for scarring and/or inflammation, whereas genotype was associated with the presence of follicles and/or inflammation, with genotype B having higher odds relative to genovar A for both variables. There was no evidence to suggest that scarring progression was associated with any of the variables tested. For the scarring progression analysis, 109 children did not have data available, due to missing surveys from the beginning and/or end timepoints (Supplementary Table S4).

### 3.4. Diversity

**Figure 4:**
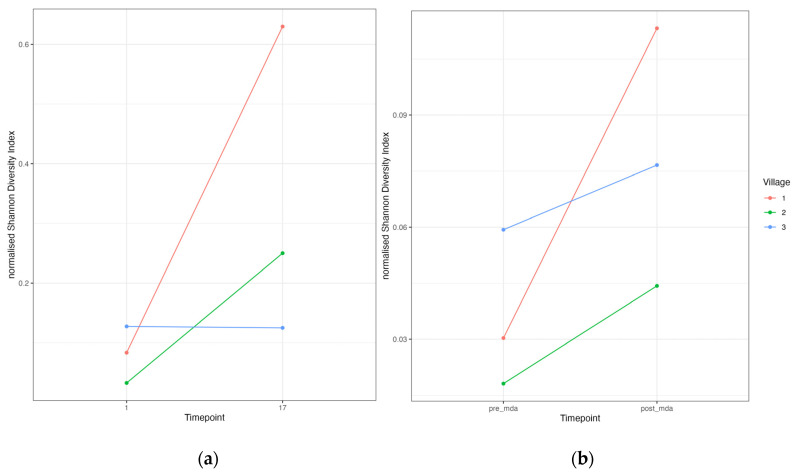
Normalized exponent of Shannon diversity separated for each Tanzanian village, comparing the first and last timepoints (**a**) and the mean diversity scores of pre-MDA (timepoints 1–3) and post-MDA (timepoints 4–17) timepoints (**b**). MDA: Mass drug administration with azithromycin.

*Ct ompA* genovariant diversity increased substantially after MDA for villages 1 and 2; however, village 3 had a much smaller increase (Figure 4). The comparison between the first and last timepoint demonstrated a similar pattern, with almost identical levels of diversity for village 3 between the two timepoints.

## 4. Discussion

The present study provides valuable insights into the dynamics of *Ct* infections, specifically focusing on *ompA* genovars and genovariants, how they are impacted by MDA, and their association with clinical signs in a trachoma-endemic region of Tanzania. The WHO has targeted trachoma for elimination for over 25 years. Progress via the SAFE strategy has been remarkable. In 2002, an estimated 1.5 billion people were at risk of trachoma; as of early 2026, that number has fallen to below 100 million—a reduction of over 90% [1]. In some areas, however, trachoma remains a significant public health concern, and understanding transmission dynamics is key to determining drivers of persistence and re-emergence in areas where SAFE has been conducted. In addition to this, despite extensive research, the absence of approved vaccines against active infection or blinding trachoma underscores the need for a deeper understanding of the factors influencing the pathogenesis of *Ct*.

Our findings highlight the prevalence and distribution of *Ct* genotypes, with a notable shift from genovar B to genovar A following the first round of mass drug administration (MDA). In a longitudinal study based in Ethiopia by Mosenia et al. [25], children who had been re-infected with *Ct* were generally re-infected with the same genovar, with only one household in 19 switching from A to B. In another study of adult Tanzanian women, over 70% were infected with the same genovar three years after the first sample collection [8]. Out of 121 people who had recurrent infections in this study, only 28 had multiple different genovariant infections over time, and of these 28, 17 switched between genovar A and B. One likely reason for the observed change from genotype B to A in this study may be partially due to the differences in genovars between the villages at baseline and the relative proportion of samples collected from each village at later timepoints. Village 3, for example, had more genovar A-positive individuals in the pre-MDA timepoints (1–3) than villages 1 and 2. As the villages are located in close enough proximity to assume some level of population mixing, it is possible that infected people from village 3 were responsible for transmitting the genovar A infections observed in villages 1 and 2 at timepoint 14 and onwards (Figure 2). Individual-level MDA coverage for the cohort was above 60% for all MDA treatments (Figure 1), and of the 121 people who were infected more than once, 114 were treated at least once prior to re-infection, over the course of the study (Supplementary Table S6). While this suggests that some people in the cohort did not receive treatment and could therefore have feasibly transmitted infection to previously recovered people, it also suggests that re-infection is a relatively common occurrence.

The study’s longitudinal design, spanning four years and incorporating multiple rounds of MDA, is a notable strength, providing a unique dataset to explore the influence of recurrent infections, genovars, and their genovariants on the prevalence of clinical signs as well as scarring progression. Association analyses between genotypes and clinical signs revealed that genotype B showed a significant association with the presence of follicles, papillae, and scarring. Genotype B has been shown to be associated with higher levels of inflammation (TI and TF) [9], and genovars A and B may trigger subtle differences in host inflammatory responses [26]. Genovar B, however, is confounded by the timing, with the vast majority of genotype B found before MDA, and the pre-MDA timepoint being significantly associated with higher follicles, papillae, and scarring. This makes it difficult to tease apart the relative roles of genovars and MDA on the severity of the clinical signs of trachoma. There were no variables that were shown to be significantly associated with scarring progression.

The longitudinal nature of this study also allows the influence of multiple infections with different genovars to be investigated. A serial genotype infection was not indicated as influential for any response variable; however, the number of people with serial infections was low, so statistical power may be lacking to confidently draw this conclusion; of the 121 people with recurrent infections, 83% were infected with the same *ompA* genovar, and 77% were infected with the same genovariant. Repeated infection even after treatment was also observed (Supplementary Table S6), suggesting the effects of MDA are relatively short-lived. A similar proportion of matching genovars was found in a study conducted on 22 children infected with *Ct* in Tanzania between 1989 and 1995 [7]. While some individuals may have been experiencing a single uninterrupted infection rather than a re-infection, this inability to clear the same or near-identical *ompA* infections indicates the hosts do not mount an immune response sufficient to clear the initial infection or prevent re-infection. While re-infection is a common factor, most likely from non-treated individuals, the stability of genotypes observed might also suggest that chlamydial persistence may serve as an alternative explanation. Experimental evidence indicates that *Ct* can transition into a reversible, non-replicative state characterized by aberrant, non-cultivable reticulate bodies when exposed to antibiotics like azithromycin [27]. This biological evasion strategy may warrant further consideration as a potential driver of the high recurrence rates.

The analysis of *ompA* genovariants within genovars revealed the dominance of a small number of genovariants, with genovariants B2 and B9 in genotype B and genovariant A2 in genovar A representing the majority of genovars. The impact of these genovariants on antigenicity was explored, demonstrating potential for significant change: Since the hydroxyl (OH) group of threonine is a stronger hydrogen bond donor/acceptor and can be a site for phosphorylation or O-linked glycosylation, this substitution can drastically alter how an antibody recognizes the residue. If the residue is on the surface, the change from non-polar (A) to polar (T) can modify the local hydrophilicity and surface accessibility, which are key factors for B cell epitope recognition. Even a small increase in the antigenic propensity scores due to the A → T change could cause the region to move from non-epitope to epitope (or vice versa), making the change meaningful for prediction. B9 was the only genovariant with a high enough frequency to allow statistical analysis to be conducted on its impact on clinical signs; while it was not significant for any of the clinical signs, the model investigating the ordinal response variable ‘follicles and/or inflammation’ did suggest that individuals infected with genovariant B9 may be up to 2.5 times more likely to have follicles and or inflammation (*p* = 0.06). The amino acid change observed in B9 switched from A → T. Threonine contains a hydroxyl (-OH) group, which can form hydrogen bonds, and can therefore significantly alter the local charge distribution and polarity within the epitope region. The OH group of threonine also has hydrogen bonding potential, and can form hydrogen bonds with other amino acids within the epitope or with other parts of the antigen; in the influenza virus haemagglutinin protein, a mutation at position 156 from Ala-to-Thr has been shown to add a glycosylation motif that blocks neutralizing antibody binding [28].

The majority of the genovariants were found in pre-MDA timepoints 1–3; however, when diversity was normalized to account for the number of infections, it was evident that diversity post-MDA was substantially higher relative to timepoints before the first round of MDA (Figure 4). This finding implies that there is an influx of *Ct* strains entering the cohort population, or incomplete control of transmission. It has been speculated that if *ompA* diversity is brought to low levels following MDA, the resulting population bottleneck of distinct chlamydial strains would result in more widespread individual-level immunity to the reduced number of genovars and hence keep the prevalence at a low level. Chin et al. [29] found there was no relationship between *Ct* diversity and *Ct* transmission; however, this study was limited by a relatively small number of positive *Ct* results per community, partly due to MDA. Others have found opposing results, where genetic diversity was significantly associated with the presence of active trachoma [10]; however, in that study, MDA was not given to the cohort. As a high proportion of repeated infection was with identical or near-identical *ompA* strains, this suggests that *ompA* diversity may be less important in overall immunity than the host’s ability to develop an effective immune response to *Ct*.

Another interesting result of this study is the observation that there was a significantly higher load of *Ct* in people infected with genovar A over genovar B. This is unexpected as in other studies genovar B was associated with higher *Ct* loads and a prevalence of clinical signs, indicating more inflammation [30,31]. However, as shown in Figure 3, *Ct* load fluctuated substantially over time for both genovar A and genovar B, with load increasing in genotype A after timepoint 6. MDA is likely to have had an influence on the community *Ct* load [32]. The limitation of using load as a factor is that studies have shown that *Ct* load can vary substantially between individuals [33] and between strains [34]. Studies have also shown that plasmid copy number in *Ct* and other *Chlamydia* species can be increased, potentially as a response to chemical stress [35].

There are several study limitations; village 3 had a much larger population sampled throughout the study, as well as an additional round of MDA. As this village appeared to be driving much of the infection with genovar A at later timepoints but did not have as high a proportion sampled at earlier timepoints where genovar B was dominant, it creates confusion with regard to the relative roles of genovar in *Ct* virulence, as well as relative genovariant diversity. Multiple rounds of MDA also made determining the effect of individual MDA on re-infection difficult; of the 76 people who experienced infection before and after treatment, there was substantial individual variation between the amount of time that had passed after the most recent round of MDA and the subsequent re-infection (Supplementary Table S4). However, this is the frequency with which MDA is delivered by the national programme for control, as advised by WHO [36], and is therefore reflective of trachoma control dynamics. Further limitations include relying solely on *ompA* for genovar classification, which may increase the risk of genovariant misclassification via sequencing artifacts or a low sensitivity to detect mixed infections. Furthermore, given that infections in 4–15-year-olds may last only two weeks [37], a three-month sampling interval, combined with potential variations in plasmid copy numbers, may not provide an ideal representation of infection dynamics.

## 5. Conclusions

The long-term study of *Ct* dynamics in a trachoma-endemic Tanzanian population illuminates the complex effects of MDA. MDA may have influenced genovar diversity by driving a shift from the dominant B genovar to the A genovar. This genovar shift may be of significance: Genovar B showed a stronger link to severe clinical signs (inflammation and scarring), while genovar A was associated with higher bacterial burden, highlighting distinct genovar–host interaction patterns. The persistent recurrence of infections, often by the same genovar after treatment, points to problems with incomplete clearance and a weak protective immune response. Ultimately, these results call for research focused on genovar-specific immune mechanisms and virulence factors to develop an effective trachoma vaccine and optimize future eradication campaigns.

## Figures and Tables

**Table 1:** Oligonucleotides used in ddPCR and qPCR diagnostic assays.

Target	Oligo	Sequence (5′-3′)	Amplicon Size (b.p)	Genomic Location	
				Start Position	End Position
*C. trachomatis omcB* (60 kDa cysteine-rich outer membrane protein) A/HAR13 (Accession: CP000038.1) Gene ID: CTA_0474	Primer (F)	GACACCAAAGCGAAAGACAACAC	105	507,750	507,772
	Primer (R)	ACTCATGAACCGGACAACCT		507,834	507,854
	Probe	[FAM]-CCACAGCAAAGAGACTCCCGTAGACCG-[QSY]		507,787	507,813
*C. trachomatis pORF2* (pgp3; Secreted effector protein; highly immunogenic and essential for plasmid maintenance. Plasmid pCT (Accession: CP000039.1)	Primer (F)	CAGCTTGTAGTCCTGCTTGAG AGA	102	1130	1153
	Primer (R)	CAAGATACATCGGTCAACGAAGA		1208	1231
	Probe	[NED]-CGGGCGATTTGCCTT-[MGBNFQ]		1168	1182
*H. sapiens RPP30* (Ribonuclease P/MRP Subunit P30) Exon 1. (Accession: NC_000010.11).	Primer (F)	AGATTTGGACCTGCGAGCG	81	90,563,013	90,563,031
	Primer (R)	GAGCGGCTGTCTCCACAAGT		90,563,074	90,563,093
	Probe	[VIC]-TTCTGACCTGAAGGCTCTGCGCG-[QSY]		90,563,041	90,563,063

b.p: base pairs; F: forward; R: reverse; *omcB*: outer membrane protein complex B; *pORF2*: plasmid open reading frame 2; *RPP30*: RNase P/MRP 30 kDa subunit. *RPP30* based on the latest Human Genome Reference GRCh38.p14. located on Chromosome 10.

**Table 2:** Oligonucleotides used in *ompA* PCR and sequencing.

Target	Oligo Primer Sequence (5′-3′)	Amplicon Size (b.p)	Genomic Location	
			Start	End
*Chlamydia trachomatis* A/HAR-13 reference genome (Accession: CP000038.1)				
First round PCR				
Target Gene: *ompA* (Gene ID: CTA_0645).	*ompA*-87 TGAACCAGCCTTATGATCGACGG	1077	702,154	702,177
	*ompA*-1163 CGGAATTGTGCATTTACGTGAG		703,209	703,230
Second round (nested) PCR				
Target Gene: *ompA* (Gene ID: CTA_0645).	*ompA*-87 TGAACCAAGCCTTATGATCGACGG	983	702,154	702,177
	*ompA*-1059 GCAAGATTTTCTAGATTTCATC		703,115	703,136

**Table 3:** Association analysis results. The reference levels for genovar, age, and gender were genotype A, 1 year, and female, respectively. Genovariant refers to the second most frequent genovar B genovariant B9 (*n* = 21). Only the results for variables that were included in the final models with the lowest AIC are shown.

Response Variable	Dependent Variables	Adjusted Odds Ratio (95% CI)	*p* Value
Scarring (0–3)	Age Gender Genotype B Genotype Ba	1.23 (0.98–1.54) 0.90 (0.37–2.15) 13.64 (4.83–37.04) 4.79 (0.17–132.8)	0.08 0.79 <0.001 0.36
Follicles (0–3)	Age Gender Genotype B Genotype Ba	0.80 (0.70–0.92) 1.32 (0.79–2.28) 2.32 (1.44–3.74) 3.45 (0.20–60.00)	0.002 0.29 <0.001 0.40
Papillae (0–3)	Age Gender Genotype B Genotype Ba	0.97 (0.81–1.14) 1.14 (0.58–2.20) 3.31 (1.83–5.96) 1.16 (0.07–19.24)	0.70 0.77 <0.001 0.92
Scarring and/or inflammation (0–2)	Age Gender Genotype B Genotype Ba	1.21 (1.05–1.40) 1.21 (0.7–2.09) 7.66 (3.52–16.71) 5.35 (0.45–63.18)	0.00858 0.5 <0.001 0.18
Follicles and/or inflammation (0–2)	Age Gender Genovariant B9 Genotype B Genotype Ba	0.95 (0.86–1.06) 1.2 (0.80–1.77) 2.53 (0.97–6.59) 2.48 (1.57–3.90) 1.92 (0.23–16.25)	0.4 0.4 0.06 <0.001 0.06
Scarring progression (Y/N)	Age Gender Serial genotype infection	0.95 (0.76–1.17) 1.35 (0.62–2.97) 1.68 (0.54–5.23)	0.608 0.449 0.371

## Data Availability

The data that were used to support the findings of this study are available from the corresponding author upon request. *Ct* WGS used in this study are available at ENA PRJEB46956. New sequences generated and used in the study are openly available at the PZ438546–PZ438994.

## Supplementary Materials

The following supporting information can be downloaded at: https://www.mdpi.com/article/10.3390/pathogens15070705/s1; Table S1: *Chlamydia trachomatis* genotype A genovariants from sequenced eye swab DNA from Tanzanian children, with the position and base pair followed by the amino acid. Reference (DQ064279) sequence used for alignment was A/HAR13; Table S2: *Chlamydia trachomatis* genotype B genovariants from sequenced eye swab DNA from Tanzanian children with the position and base pair followed by the amino acid. Reference (M17342) sequence used for alignment was B/TW-5; Table S3: Number of Tanzanian children presenting the clinical signs follicles (F), papillae (P) and scarring (S) per *Chlamydia trachomatis* genotype and village; Table S4: Demographic table of Tanzanian children excluded from the analysis due to not having information on scarring progression in comparison to those included- age, sex, village. TP: Trachomatous papillae. TF: trachomatous; Table S5: Number of Tanzanian children with each *Chlamydia trachomatis* (*Ct*) genotype per study timepoint. NA represents Ct positive samples that were not able to be genotyped; Table S6: Infection and treatment with azithromycin timepoints for Tanzanian children who were positive for Chlamydia trachomatis more than once (N = 120).

## Abbreviations

The following abbreviations are used in this manuscript:

MDA	Mass drug administration
SNV	Single-nucleotide variant
WHO	World Health Organization
SAFE	Surgery, antibiotic, facial cleanliness, environmental improvement
FPC	Follicles, papillae, and cicatrices
MOMP	Major Outer Membrane Protein
*ompA*	Outer membrane protein A
S	Conjunctival scarring
