# Supplementary material for: Chlamydia trachomatis ompA Genotype and Clinical Signs of Trachoma in a Longitudinal Tanzanian Cohort
Source: Pathogens. 2026 Jul 4;15(7):705. doi: 10.3390/pathogens15070705 (PMC13414724; doi:10.3390/pathogens15070705)
Supplement: Supplementary file 1 [file pathogens-15-00705-s001.zip › pathogens-4134124-supplementary.pdf]

Table S1. *Chlamydia trachomatis* genotype A genovariants from sequenced eye swab DNA from Tanzanian children, with the position and base pair followed by the amino acid. Reference (DQ064279) sequence used for alignment was A/HAR13.

Ref A/HAR	240A 80(K)	304G 102	375T 125	446A 149	523C 175	571A 191(D)	582T 194	627T 209	795G 265	843T 281	873T 291(I)	992C 330
A0 N=1	As reference											
A1 N=1	A-> G (K)											
A2 N=213		G->A (T)			C->A (L)							
A3 N=1			T->C (A)	A->G (R)								
A4 N=1						G>A (N)						
A7 N=1										T>C (S)		

Table S2. *Chlamydia trachomatis* genotype B genovariants from sequenced eye swab DNA from Tanzanian children with the position and base pair followed by the amino acid. Reference (M17342) sequence used for alignment was B/TW-5.

**Ref B/TW-5**	**212A (D)**	**216C (R)**	**218T (T)**	**226A (T)**	**268A (T)**	**287T (A)**	**347C (A)**	**484A (N)**	**496A (T)**	**511A (S)**	**514G (A)**	**585T (A)**	**594G (A)**	**597T (R)**	**741A (E)**	**797C (S)**	**917C (A)**	**927G (K)**	**955A (T)**	**1000G (A)**
B1 n=1	A->T (V)																		A->G (A)	
B2 n=194		C->T (R)			A->G (A)	T->C (G)				A->G (G)	G->A (T)	T->C (A)	G->T (A)	T->C (R)	A->G (E)					
B3 n=1			T->C (A)	A->G (A)																
B5 N=1								A->G (D)												
B6 N=1									A->G (A)											
B7 N=1																C->T (F)		G->A (K)		
B8 n=1																	C->T (V)			
B9 n=21																				G->A (T)

Table S3. Number of Tanzanian children presenting the clinical signs follicles (F), papillae (P) and scarring (S) per *Chlamydia trachomatis* genotype and village.

**Genotype**	**Village 1**			**Village 2**			**Village 3**		
	**F**	**P**	**S**	**F**	**P**	**S**	**F**	**P**	**S**
A	10	8	2	3	2	0	155	96	9
B	102	78	46	40	30	13	50	38	14
Ba	3	2	1	0	0	0	0	0	0

Table S4. Demographic table of Tanzanian children excluded from the analysis due to not having information on scarring progression in comparison to those included- age, sex, village. TP: Trachomatous papillae. TF: trachomatous

**Variable**	**Included in analysis**	**Excluded from analysis**
Total number of samples	340	109
Mean age (range)	10.2 (8-15)	10.7 (8-15)
Gender (% female)	63	51
Village 1 (n)	84	50
Village 2	44	10
Village 3	212	49
Mean Infection proportion	23.9	33.8
Mean TP proportion	14.6	21.3
Mean TF proportion	25.4	33.3
Mean TI proportion	6.3	8.6
Mean AT proportion	4.3	6.2

Table S5. Number of Tanzanian children with each *Chlamydia trachomatis (Ct)* genotype per study timepoint. NA represents Ct positive samples that were not able to be genotyped.

	**Time point**																	
**Genotype**	**1**	**2**	**3**	**4**	**5**	**6**	**7**	**8**	**9**	**10**	**11**	**12**	**13**	**14**	**15**	**16**	**17**	**Total**
A	3	7	0	0	0	0	7	3	6	25	30	14	24	38	36	13	14	220
B	67	56	38	5	11	4	4	4	4	5	9	0	1	3	5	1	5	222
Ba	1	5	1	0	0	0	0	0	0	0	0	0	0	0	0	0	0	7
NA	7	15	15	1	1	4	10	10	2	21	6	11	8	18	8	5	2	143
Total	78	82	54	6	12	8	21	17	12	51	45	25	33	59	49	19	21	591

Table S6. Infection and treatment with azithromycin timepoints for Tanzanian children who were positive for *Chlamydia trachomatis* more than once (N=120).

**Id**	**Infection Timepoints**	**Genovariants**	**Treated At 1st Round**	**Treated At 2nd Round**	**Treated At 3rd Round**	**Infection After Treatment**
111321	4,5,7	B2,B2,B2	No	Yes	Yes	No
113165	1,2,3	B2,B2,B2	Yes	No	Yes	No
126412	14,15,16,17	B2,B2,B2,B2	No	Yes	No	Yes
129141	2,3	B2,B2	Yes	Yes	No	No
129164	5,6,7	B2,B2,Na	No	No	Yes	No
129169	1,2,3	B2,B2,B2	Yes	Yes	Yes	No
129412	1,2,3	B2,B2,B2	Yes	No	Yes	No
129421	2,3,4	Na,B2,B2	Yes	Yes	Yes	No
129432	2,3	B2,B2	Yes	No	Yes	No
129433	14,16,17	A9,Na,A2	Yes	Yes	Yes	Yes
129513	14,15,16	A2,Na,A2	Yes	No	Yes	Yes
13151111	1,2,3	B2,Na,B2	Yes	Yes	No	No
13151112	1,2,3	B2,B2,Na	Yes	Yes	Yes	No
13151115	1,2,3	B2,B2,Na	Yes	No	Yes	No
1315114	1,2	B2,B2	Yes	Yes	Yes	No
1315119	1,2,3	B2,B2,B2	Yes	Yes	Yes	No
211613	1,2,3,5	B2,B2,Na,B2	Yes	Yes	Yes	Yes

211711	1,3	B2,B2	Yes	Yes	Yes	No
211712	3,5,6,8	B2,B2,Na,B2	Yes	Yes	Yes	Yes
211735	1,2	B2,B2	No	No	No	No Treatment
211739	2,6	B2,B2	No	No	No	No Treatment
212111	1,2,6	B2,B2,Na	No	No	Yes	No
212112	1,2,3,16	B2,B2,B2,A2	Yes	Yes	No	Yes
2121510	1,4,5,6,7	B2,B2,B2,B2,Na	No	Yes	Yes	No
2121514	16,17	A2,A2	Yes	Yes	Yes	Yes
212152	2,3	B2,B2	Yes	No	No	No
212155	16,17,19	A2,A2,A2	Yes	Yes	Yes	Yes
212159	1,2	B2,B2	Yes	No	No	No
213131	1,2	B2,B2	No	No	Yes	No
213171	3,9,10	B2,B2,B2	Yes	No	Yes	Yes
2131711	1,2,3	B2,Ba,Ba	Yes	No	Yes	No
2131719	2,7	B2,B2	No	No	Yes	No
213174	2,9,10	Na,B2,A2	Yes	No	Yes	Yes
213176	1,2,3	B9,B9,B9	Yes	No	No	No
213179	1,11	B2,B4	Yes	No	Yes	Yes
215112	1,2,3,16	B2,B2,B2,A2	Yes	Yes	Yes	Yes
215121	15,16	A2,A2	Yes	Yes	Yes	Yes
215141	1,3,5	B2,B2,Na	Yes	Yes	Yes	Yes
215143	16,17	A2,A2	Yes	Yes	Yes	Yes
215151	2,5,6	Na,B2,B2	No	No	No	No
215164	1,2,3,16	B2,B2,Na,A2	Yes	Yes	Yes	Yes
215213	1,2,3,7	B2,B2,B2,Na	Yes	Yes	Yes	Yes
216111	1,2	B2,B2	Yes	Yes	Yes	No
216113	1,2,3	B2,B2,B2	No	Yes	Yes	No
216122	1,10	B2,B6	Yes	Yes	Yes	Yes
216123	1,2,3	B2,A0,B2	Yes	Yes	Yes	No
216154	2,7	B2,B2	No	Yes	Yes	No

219113	10,17	B2,B2	Yes	No	Yes	Yes
219131	1,2,8,9	B2,B2,B2,B2	No	No	Yes	No
219512	1,2,3,17	B2,B2,B2,B2	Yes	Yes	Yes	Yes
219614	1,2	B2,B2	Yes	No	Yes	No
311122	2,10	B2,A2	No	No	No	No Treatment
311322	10,11	A2,A2	Yes	No	Yes	Yes
312131	14,15	A2,A2	No	No	Yes	Yes
312143	14,16,17	A2,A2,A2	No	No	Yes	Yes
313111	11,12,13,14	Na,A2,Na,A2	Yes	No	Yes	Yes
313122	10,11	A2,A2	No	No	Yes	No
313142	10,11,13,14,15	Na,A2,A2,A2,Na	No	No	Yes	Yes
313143	11,12,14	A2,A2,A2	Yes	No	No	Yes
313161	14,15,16,17	A2,A2,Na,A2	No	No	No	No Treatment
313171	10,11,12,13,14,15,16,17	Na,A2,Na,A2,A2,A2,A2,A2	Yes	No	Yes	Yes
313212	12,13,14,15	A2,A2,A2,A2	No	No	Yes	Yes
313231	10,14,15	A2,B2,B2	Yes	No	Yes	Yes
313232	10,11,14,15	A2,A2,B2,B2	Yes	Yes	Yes	Yes
314142	12,13,14,15	A2,Na,A2,Na	Yes	No	No	Yes
314152	10,14,15	A2,A2,A2	No	No	Yes	Yes
314171	12,13,14,15	A2,A2,A2,A2	Yes	Yes	Yes	Yes
314172	12,13,14,15	A2,A8,A2,A2	Yes	Yes	Yes	Yes
314411	7,10,11	Na,A2,A2	Yes	No	Yes	Yes
314412	8,10,11	Na,A2,A2	Yes	Yes	Yes	Yes
314421	7,8,9,10,11	A2,A2,A2,A2,A2	Yes	No	Yes	Yes
314431	9,10,11,15	A2,A2,A2,A2	Yes	Yes	Yes	Yes
314451	7,8,9,10,11	Na,Na,A2,A2,A2	Yes	Yes	Yes	Yes
314461	7,8,9,11,14	A2,A2,A2,A2,Na	Yes	Yes	Yes	Yes
314471	7,8,10,14,15	A2,Na,A2,A2,Na	Yes	No	Yes	Yes
314481	7,8,10,11,14,15,16	A2,B1,Na,A2,A2,A2,A2	Yes	No	Yes	Yes

314521	10,11	A2,A2	Yes	No	Yes	Yes
3310112	1,2,7,11,12,13,14	A2,A2,A2,A2,Na,A2,Na	Yes	No	No	Yes
3310151	2,13,14	A2,A2,A2	Yes	Yes	Yes	Yes
3310152	10,11,12,13	A2,Na,Na,A2	No	No	Yes	Yes
3310153	2,11,12,13,14	A4,A2,A2,A2,A2	Yes	No	Yes	Yes
3310171	1,2	A2,A2	No	No	No	No Treatment
3310192	1,2	A2,A2	Yes	No	No	No
3311113	11,12,13,14,15	A2,Na,B2,A2,A2	Yes	Yes	Yes	Yes
3311141	13,14	A2,A2	Yes	No	No	Yes
3311162	13,14	A2,A2	Yes	No	No	Yes
3311241	13,15	A2,A2	Yes	No	Yes	Yes
3311252	14,15	A2,A2	Yes	No	Yes	Yes
3313121	14,15	A2,A2	Yes	No	Yes	Yes
3313134	2,4	B2,B2	Yes	No	Yes	Yes
3313311	1,2,3	Na,B2,B2	Yes	Yes	Yes	No
3313312	1,2,3,6,15,17	B2,B2,Na,Na,A2,A2	Yes	Yes	Yes	Yes
3314113	1,2,14,15,17	B2,B2,A2,A2,A2	Yes	Yes	Yes	Yes
3314114	1,2	B2,B2	Yes	Yes	Yes	No
3314115	1,2,3,14,15	Ba,B2,B2,A2,A2	Yes	Yes	Yes	Yes
3314221	1,2,14	B2,B2,Na	Yes	No	Yes	Yes
3314226	14,15	A2,A2	Yes	Yes	Yes	Yes
3314227	2,13,14,15	A2,A2,Na,A2	Yes	Yes	No	Yes
3315112	14,15	A2,A2	Yes	No	Yes	Yes
3315113	13,14,15	A2,A2,A2	Yes	No	Yes	Yes
3315223	13,14,15	A2,A2,Na	Yes	No	No	Yes
3315233	11,12,13,14	A2,A2,A2,A2	Yes	No	No	Yes
3315242	11,12,13	A2,A2,A2	Yes	No	Yes	Yes
3371103	1,12,13,14	B9,A2,A2,A2	No	No	No	No Treatment
337111	10,11,12,13	A2,A2,A2,A2	No	No	Yes	Yes

337121	3,10,11	Na,A2,A2	Yes	No	Yes	Yes
337171	3,10,11	B9,A2,A3	Yes	No	Yes	Yes
337181	1,2,3	B2,B2,Na	Yes	No	Yes	No
337182	1,2	B9,B9	Yes	No	Yes	No
3381131	1,2,3,14	B2,B2,B2,Na	Yes	Yes	Yes	Yes
3381142	1,3,9,12	B2,B2,A2,A2	Yes	No	Yes	Yes
3381183	13,14,15	A2,Na,A2	Yes	No	Yes	Yes
3381201	1,2,3,11	B9,Na,B9,A2	Yes	No	Yes	Yes
339113	1,15	B9,A2	Yes	Yes	Yes	Yes
339131	1,2,3	B9,Ba,Na	Yes	No	Yes	No
339151	1,3	B9,B9	Yes	No	Yes	No
339171	1,2,3,10,17	B2,Na,B9,A2,A2	Yes	No	Yes	Yes
339181	1,2,3,16,17	B9,B9,B9,Na,A2	Yes	No	Yes	Yes
339262	1,2	B9,B9	No	No	Yes	No
339281	1,2,9,10,11,12,13,14,15	Na,B2,A2,A2,A2,A2,A2,Na,Na	Yes	No	Yes	Yes
